# Evaluating Habitat Suitability for the Establishment of *Monochamus spp.* through Climate-Based Niche Modeling

**DOI:** 10.1371/journal.pone.0102592

**Published:** 2014-07-14

**Authors:** Sergio A. Estay, Fabio A. Labra, Roger D. Sepulveda, Leonardo D. Bacigalupe

**Affiliations:** 1 Instituto de Ciencias Ambientales y Evolutivas, Facultad de Ciencias, Universidad Austral de Chile, Valdivia, Chile; 2 Centro de Investigación e Innovación para el Cambio Climático, Facultad de Ciencias, Universidad Santo Tomas, Santiago, Chile; Natural Resources Canada, Canada

## Abstract

Pine sawyer beetle species of the genus *Monochamus* are vectors of the nematode pest *Bursaphelenchus xylophilus.* The introduction of these species into new habitats is a constant threat for those regions where the forestry industry depends on conifers, and especially on species of *Pinus*. To obtain information about the potential risk of establishment of these insects in Chile, we performed climate-based niche modeling using data for five North American and four Eurasian *Monochamus* species using a Maxent approach. The most important variables that account for current distribution of these species are total annual precipitation and annual and seasonal average temperatures, with some differences between North American and Eurasian species. Projections of potential geographic distribution in Chile show that all species could occupy at least 37% of the area between 30° and 53°S, where industrial plantations of *P. radiata* are concentrated. Our results indicated that Chile seems more suitable for Eurasian than for North American species.

## Introduction

Currently, there are hundreds to thousands of exotic species established outside their native ecosystems [1]. Probably these numbers will increase in the future as a result of the steady growth in international trade which produces human-aided long-distance dispersal of organisms [2].

Forests in Asia, Europe and North America have experienced the introduction of insect pests which have caused ecological, social and economic damage to natural forest, industrial plantations and urban trees. Given that eradication of established invasive species often implies large economic costs concurrent with a low probability of success, the logical recommendation for governments is to place the highest priority on preventing introduction of such species [3]–[4]. In this regard, pest risk assessment (PRA) is a key procedure that encompasses several methodologies that aim to evaluate the likelihood of an exotic species being introduced to a region and causing damage to agriculture [5]. Thus, PRA uses biological and economic information to determine whether some species should be regulated and the strength of the sanitary measures to be taken against it [6].

One of the necessary steps of a PRA is the assessment of the suitability of the new habitat for the establishment of the exotic organism [6]–[8]. Over the last decade, ecologists have developed several tools with solid bases in mathematics, statistics and information theory that facilitate these analyzes [4], [9]–[12]. Among these, climate-based ecological niche modeling is commonly used in risk assessment [13]–[14]. Climate-based ecological niche models may be considered as a subset of the more general species distribution models, which are numerical tools that combine observations of species (either presences or presences and absences) in a set of locations with environmental variables to obtain ecological and evolutionary insights and to predict distributions across landscapes [11], [15]. In recent years, niche models have been used to predict potential geographic distribution of several forest pests such as the Asian longhorn beetle [16], pine shoot beetle [17], European woodwasp [18], redbay ambrosia beetle [19] and emerald ash borer [14].

One of the most serious threats to pine forests in the world is pine wilt disease, caused by the pinewood nematode, *Bursaphelenchus xylophilus*. This disease is native to North America where it is a secondary pathogen of native pines, but is the cause of pine wilt disease in non-native pines [20]. In countries where the pinewood nematode has been introduced, such as Japan, pine wilt is an important non-native disease [21]–[22], with estimated losses of 46 million m^3^ of wood in the last 50 years [23]. Although this nematode may be carried by several xylophagus insects, successful transmission to conifers has only been demonstrated for the pine sawyer beetles of the genus *Monochamus* [24]–[25].

There are no native species of *Monochamus* in South America and they are included in the list of insects recommended for regulation as quarantine pests of the COSAVE (Regional plant protection organization of the Southern Cone of South America). The potential introduction of these species to a continent where *Pinus* plantations are a key component in the forest industry [26] could have serious economic consequences. In the case of Chile, commercial plantations of *P. radiata* are the basis for the forestry industry. Currently, Chile has 1.5 million ha of *P. radiata* plantations established across several site types and climate conditions that vary from 30° to 43°S latitude [27]. In addition, urban trees of this species as well as of other *Monochamus* hosts (*Picea*, *Abies*, *Cedrus* and *Pseudotsuga*) may be found in most Chilean cities all over the country. In this study we used ecological niche modeling methods to obtain insights on the role of climate in shaping the current distribution of nine species of *Monochamus* vectors of *B. xylophilus* and the relative importance of each variable analyzed in determining native geographic ranges for each species. We then use these models to generate a map of the potential distribution of each of these species in Chile, which may be used as a proxy of the suitability of the new habitat in a PRA.

## Materials and Methods

### Species occurrence

Records of confirmed presences (i.e. confirmed establishment) of *Monochamus* species were obtained from multiple primary sources. The primary sources used were the open databases Invasive Species Compendium [28] and the EPPO Plant Quarantine Data Retrieval System (PQR, [29]). Both datasets are considered within the PRATIQUE initiative of EPPO [30]. To complement this information, we also used information from Dillon and Dillon [31] and Cherepanov [32] for North American and Eurasian *Monochamus,* respectively. When no geo-referenced localities (just locality names) were provided, geographic coordinates were obtained from official gazetteers (GeoNet, [33]; TGN, [34]). We restricted our study to species with at least 20 confirmed records. These procedures allowed us to obtain datasets for five North American species, and four Eurasian species. The species considered and the respective number of data points were as follows. In North America: *M. carolinensis* (34), *M. marmorator* (25), *M. notatus* (36), *M. scutellatus* (47), *M. titillator* (39). For Eurasia: *M. alternatus* (32), *M. galloprovincialis* (49), *M. saltuarius* (24) and *M. sutor* (47) (Table S1–S2). All these species are either known to be vectors of *B. xylophilus* or are considered potential vectors [24]–[25]. All confirmed records were used, making no difference between native and exotic distributions [35]–[40].

### Climatic variables

Current global climatic conditions grids with a spatial resolution of 2.5 arc-minutes were obtained from the WorldClim database [41]. These grids contain variables compiled from monthly data collected from 1950 to 2000. Based on the biological knowledge about these species [24]–[25], [42]–[43], we selected six ecologically relevant bioclimatic variables: annual mean temperature, mean temperature of the coldest quarter, mean temperature of the warmest quarter, annual accumulated degree days (base 5°C), mean relative humidity and total annual precipitation. The “coldest” and “warmest” quarter are defined according to the Worlclim database: the mean temperature of the three-months period with the lowest and highest average temperature, respectively. We also incorporate altitude as a descriptor of topography to obtain seven explanatory variables in our modeling procedure (Table 1).

**Table 1:** Ranges of the environmental variables observed into the 95% geographic kernel defined for each species.

					Environmental variables			
Region	Species	Ann T (°C)	T°Col (°C)	T° War (°C)	ADD	% RH	PP (mm)	Altitude (masl)
	*M. carolinensis*	−5.7–25.5	−22.8–20.8	7.6–30.5	0–7190	0–81.5	192–1970	−6–3625
	*M. marmorator*	−5.7–19.2	−24.2–11.0	9.7–27.0	0–4891	0–81.5	393–1970	−6–1294
**North America**	*M. notatus*	−7.1–20.9	−26.0–14.0	4.0–28.8	0–5605	0–86.4	192–3098	−6–3625
	*M. scutellatus*	−16.1–25.1	−32.5–21.7	−12.0–33.4	0–7051	0–86.4	51–3573	−88–3748
	*M. titillator*	−2.5–26.2	−19.3–23.8	7.5–30.4	0–7562	0–80.7	205–1970	−6–3625
	*M. alternatus*	−11.5–28.1	−25.5–26.5	−4.3–30.5	0–8448	0–83.3	0–5576	−2–6512
**Eurasia**	*M. galloprovincialis*	−23.2–28.0	−49.4–18.0	−2.5–37.6	0–7707	0–90.3	0–2718	−416–3355
	*M. saltuarius*	−23.2–21.9	−49.4–15.7	−2.5–29.1	0–5240	0–90.3	0–2953	−51–5909
	*M. sutor*	−23.2–19.6	−49.4–12.5	−2.5–31.3	0–5031	0–90.3	0–2838	−41–6098
**Chile**	**Pinus Plantations**	−5.0–17.4	−9.4–12.8	−0.6–22.4	0–3346	0–86.8	0–3073	0–4339

Ann T° = mean annual temperature, T°Col = mean temperature of the coldest season, T° War = mean temperature of the warmest season, ADD = annual accumulated degree-days, % RH = annual mean relative humidity and PP = total annual precipitation. Pinus plantations refers to the area of Chile covered with *Pinus radiata* plantations (see methods for details).

### Modeling methods

Because of our datasets were based on presence-only localities, we used a maximum entropy modeling approach to estimate climate-based niche models for all 9 species. Analysis was performed with the Maxent 3.3.3 k software [44]–[48]. Comparison of the prediction accuracy across several niche modelling methods showed Maxent to be among the best modeling approaches for presence-only data [48]. Briefly, Maxent is a machine-learning algorithm that works by minimizing the relative entropy of the probability densities calculated from the presence records versus those probability densities were calculated from random sampling over the study region [44], [46]–[47]. It is important to note that Maxent is a density estimation method, and not a regression method, and as such it has properties that make it robust to limited amounts of training data (small samples) [11], [45]. Also, its results are less affected by variable autocorrelation and it allows flexible modeling of different types of functions between environmental variables and the probability of species occurrence [44].

We examined the output of the fitted model in logistic format, to indicate the suitability of the habitat of each species in the landscape. The study area to fit the model was restricted to the 95% spatial kernel for North America and Eurasia according the current registered presence of each species. Models were then evaluated using area under the curve (AUC) of the Receiver operating characteristic (ROC) curve and regularized training gain. The ROC curve corresponds to the plot between 1-specificity (proportion of false positives) versus sensitivity (proportion of true positives, [45]). The AUC index measures the ability (probability) of the maxent model to discriminate between presence sites versus background sites [44], [49]–[50]. To complement the model evaluation by AUC values, we also used regularized training gain (hereafter gain), which corresponds to the logarithm of the average ratio between the likelihood assigned to an observed presence site and the likelihood assigned to a background site. The observed value of gain was also used to estimate the relative importance of each variable by using a jackknife method. Briefly, the decrease in gain by fitting a model using all variables except the focal one was compared with the gain of the previously full model (including all variables). Next, we fit a model using only the focal variable and compared the gain in relation to the full model. This procedure yielded an estimate of the relative importance of each variable in the model. Modeling results to a 20-fold cross-validation scheme considering the usual highly correlation between climatic variables [50]–[51]. This cross-validation scheme divides the dataset into 20 subsets. In each step the model is fitted using 19 subsets and using the last one (independent) to test (validate) the fitting. This procedure is repeated 20 times, and the AUC and jackknife values reported correspond to the average value of the 20 testing procedures.

Fitted models of each species were later projected over the continental Chilean territory using the same environmental variables described previously. Given the logistic scale used, these maps may be interpreted as a measure of the suitability of the habitat (0 = unsuitable, 1 = highly suitable) and are a proxy of how favorable the habitat is for the establishment of these pests. To estimate the extent of Chilean territory these species could occupy, original logistic maps were converted to binary maps (0 = absence, 1 = presence) applying a threshold that maximizes test sensitivity and specificity [52]. These binary maps were projected on Chilean territory and on the proportion of territory covered by *Pinus* plantations. The percentage of all territory and *Pinus* plantations potentially covered for each species was calculated. Area of *Pinus* plantations was obtained using the VII national agricultural, livestock and forestry census [53]. This map corresponds to agricultural districts that contain at least one commercial *Pinus* plantation.

Manipulation of environmental layers was performed in R environment [54], Quantum GIS 1.8.0 [55] and GRASS 6.4.2 [56].

## Results

All fitted models showed high values of AUC, which makes us confident of a high discriminative ability. The lowest AUC (0.64) was obtained for the North American *M. titillator*, while the highest (0.77) was obtained for the Eurasian *M. saltuarius* (Table 2).

**Table 2:** Jacknife statistics of model performance and relative importance of each variable.

							Environmental variables			
Region	Species	AUC	Gain	Ann T°	T°Col	T° War	ADD	% RH	PP	Altitude
	*M. carolinensis*	0.73	0.283	0.141−0.036	0.121−0.043	0.154−0.000	0.151−0.044	0.141−0.070	0.102*−0.123^†^	0.141−0.070
	*M. marmorator*	0.70	0.362	0.275−0.103	0.281−0.146	0.232−0.070	0.268−0.098	0.276−0.016	0.227*−0.201^†^	0.276−0.016
**North America**	*M. notatus*	0.65	0.326	0.134−0.063	0.129−0.056	0.111−0.058	0.138−0.050	0.134−0.000	0.072*−0.168^†^	0.133−0.000
	*M. scutellatus*	0.74	0.740	0.433−0.140	0.456−0.147	0.414−0.117	0.420−0.123	0.433−0.134	0.307*−0.404^†^	0.434−0.134
	*M. titillator*	0.64	0.249	0.071−0.045	0.096−0.038	0.052*−0.081	0.058−0.037	0.060−0.080	0.106−0.148^†^	0.059−0.080
	*M. alternatus*	0.72	0.414	0.207*−0.305^†^	0.305−0.259	0.268−0.118	0.290−0.119	0.271−−0.046	0.236−0.246	0.271−−0.046
**Eurasia**	*M. galloprovincialis*	0.66	0.406	0.059−0.117	0.140−0.002	0.087−0.041	0.129−−0.057	0.055−−0.026	−0.026*−0.195^†^	0.055−−0.026
	*M. saltuarius*	0.77	0.671	0.329−0.148	0.460−0.064	0.447−0.039	0.442−−0.012	0.396−0.034	−0.040*−0.461^†^	0.391−0.034
	*M. sutor*	0.72	0.368	0.033*−0.088	0.138−0.043	0.137−−0.023	0.083−−0.025	0.125−−0.073	0.035−0.215^†^	0.126−−0.073

For each species, the table shows the area under the curve (AUC) and regularized training gain (Gain). For each variable first value correspond to the gain of a model fitted using all variables except the focal one. The more important variable according to this criterion is marked with *. The second value corresponds to the gain of a model fitted using just the focal variable. The more important variable according to this criterion is marked with ^†^, (see methods for details). Abbreviations as in table 1.

In general, models fitted using all variables except the focal one, showed that the exclusion of total annual precipitation and mean temperature of the warmest season caused the highest reduction in gain (Table 2). The analysis of models including just one variable showed that models fitted using total annual precipitation, mean temperature of the coldest season and mean annual temperature reached the highest gain (Table 2).

When we separate North American and Eurasian species, some differences appear. Models excluding the focal variable showed that for North American species (Fig. S1–S5) the exclusion of total annual precipitation caused the highest reduction in gain, but for Eurasian species (Fig. S6–S9) the highest reduction is caused by total annual precipitation and annual mean temperature (Table 2). On the other hand, using one variable, North American and Eurasian models showed that the variable with the highest gain was total annual precipitation in almost all species (Table 2).

**Figure 1:**
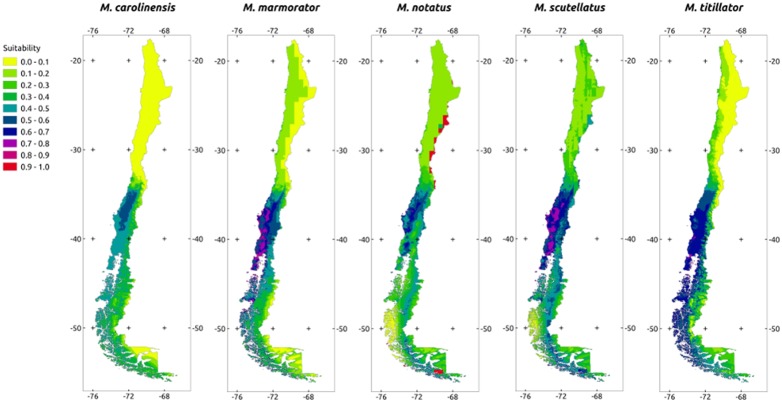
Projections of the Maxent model fitted for each North American species into Chile. Colors represent the probability of each pixel being a suitable habitat for the corresponding species.

**Figure 2:**
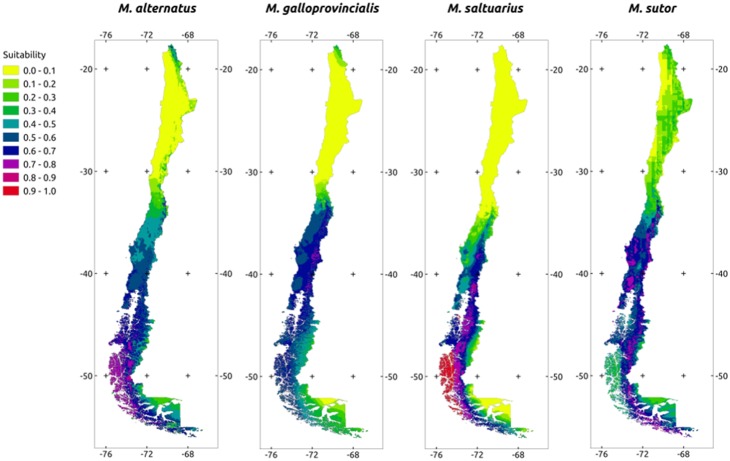
Projections of the Maxent model fitted for each Eurasian species into Chile. Colors represent the probability of each pixel being a suitable habitat for the corresponding species.

Projections of the models into the Chilean territory showed that climate in this region is moderately to highly suitable for most species (Fig. 1, 2). Specifically, the central and southern regions (35°–55°S) of Chile seem more suitable for the establishment of *Monochamus* species than the northern region (18°–35°S). The proportion of territory corresponding to suitable and unsuitable habitat showed a clear distinction between species. For North American species the main proportion of suitable habitat is between 35° to 44°S, but for Eurasian species it occurs from 35° to 56°S (Table 3).

**Table 3:** Percentage of potential area covered for each species.

Region	Species	Threshold	% All	% *Pinus*
	*M. carolinensis*	0.442	16.6	61.0
	*M.marmorator*	0.514	21.7	64.7
**North America**	*M.notatus*	0.510	13.9	37.1
	*M. scutellatus*	0.522	22.4	72.4
	*M. titillator*	0.494	32.2	71.9
	*M. alternatus*	0.472	43.9	72.5
**Eurasia**	*M. galloprovincialis*	0.430	45.9	95.5
	*M. saltuarius*	0.446	36.0	54.1
	*M. sutor*	0.473	46.0	92.6

Threshold is the logistic threshold applied to obtain a binary map. This threshold correspond to the value that maximize test sensitivity plus specificity. % All is the percentage of Chilean territory that could be potentially covered by the species. % Pinus is the percentage of territory covered by *Pinus* plantations that could be potentially covered by each *Monochamus* species.

## Discussion

In this study, we performed climate-based niche modeling for five North American and four Eurasian *Monochamus* species. Interestingly, most models showed an acceptable discriminatory power (>0.7, [49]). However, average values of AUC for North American and Eurasian species were very similar (Table 2), suggesting that model quality was equivalent between regions.

The relative importance of each variable for the fitted models showed that total annual precipitation is commonly the most important variable for species of North America and Eurasia. The decrease in gain by excluding this variable represents the amount of information provided by the excluded variable that is not present in other variables and is lost in the model by excluding it. The same situation appears in the results of models fitted using just one variable. For both regions total annual precipitation is again the most important variable. Hence, this variable could be considered as providing the highest amount of information, independently if this information is contained or not in other variables.

To the best of our knowledge, the greater importance of precipitation over temperature in conditioning the distribution of *Monochamus* species is an unexpected result. In a recent study, Chen et al. [57] pointed out that precipitation is important for the population dynamics of *M. alternatus*, but only as a secondary variable and less important than temperature. One potential explanation could be related to the link between precipitation and the distribution of host trees [58] or the influence of water content of the soil on the incidence of the symbiont nematode *B. xylophilus* [59].

Mean temperature of the warmest season, mean temperature of the coldest season and mean annual temperature are all indicators of the thermal restrictions that an organism experiences in the field. Thermal restrictions for completing development and lower thermal developmental threshold have been described for North American (*M. carolinensis* [60]) and Eurasian *Monochamus* (*M. alternatus* [42]; *M. saltuarius* [61] and *M. galloprovincialis* [62]). Ma et al. (2006) [43] even propose the −10°C January mean temperature isotherm as the northern limit of *M. alternatus* potential distribution in China. Therefore, the inclusion of these variables in our models is not surprising. However, considering the importance of thermal requirements of ectotherms we expected a higher importance of accumulated degree days, but this variable had little influence in most species.

When models were projected into Chilean territory two important results arise. First, there are important differences in the potential suitable habitat between species. On average climate in Chile seems to be more suitable for Eurasian species than for North American species, especially in the area covered for *Pinus* plantations. The reasons behind these differences may be related to the range in climatic conditions experiences by each species in its native range. In general, North American species show a more restricted distribution than the Eurasian species analyzed [28]–[29], [31]–[32].

**Figure 3:**
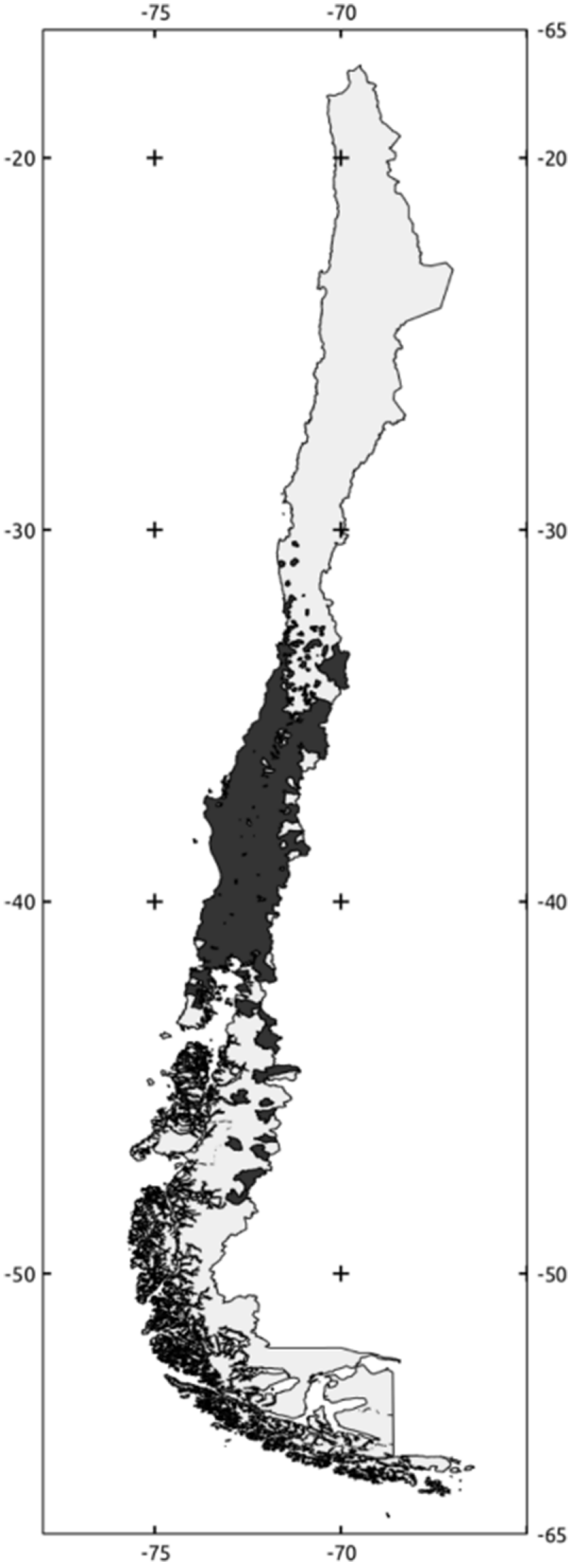
Agricultural districts of Chile that contains at least one commercial plantation of *P. radiata* (dark areas, www.odepa.cl).

*Pinus* plantations in Chile are primarily *P. radiata,* a species with controversial evidence about susceptibility to the pine wilt disease. In its native distribution, a survey performed in 1988 found no evidence of infection [63]. However, Furuno et al [64] reported approximately 80% mortality of *P. radiata* due to pine wilt disease in Japan in a 30-year experiment. Due to the contradictory evidence, EPPO classify *P. radiata* as a moderately susceptible species to *B. xylophilus* [24]. Our results show that areas with the highest probability of being suitable for *Monochamus* species are located in Central and Southern Chile mainly between 30° and 53°S. However, commercial plantations in Chile are restricted to 30°–43°S (Fig. 3). The area between 45°S and 53°S is composed mainly of conservation areas with native forest (national parks), and therefore, this region could be considered at low risk of *Monochamus* establishment. However, the region between 30° and 43°S could be considered to be at moderate to high risk of establishment of *Monochamus* (Fig. 3), if enough individuals arrives. Also, this region is the more populated part of the country and contains a high number of terrestrial, aerial and maritime ports where several interceptions of *Monochamus* have occurred in the past [65]. The combination of several potential points of introductions due to ports (high propagule pressure) and highly suitable habitat (high probability of introduction) suggest that efforts for early detection of these species should be concentrated in this region. However, it is necessary to note that low suitability habitat or low probability of establishment does not mean zero risk, and reasonable monitoring levels as well as preventive activities should be carried out even outside the 30°S–43° region.

Climate-based niche modeling has proved to be useful in forecasting the potential distribution of pest species, especially in the initial phase of a risk assessment. However, the addition of complementary distributional information (e.g. real absences) and variables other than climatic ones will reduce uncertainty in long-term risk assessment. Difficulties in the interpretation of correlative models (such as MaxEnt) have been previously highlighted [66]–[67]. Correlative models seems to be sensitive to the training data set and the addition of new information (new presences from new habitats) could caused increases in the sensitivity of the model (detection of true positives) jointly with increases of estimated prevalence [67]. In our case, the absence of independent data sets impedes the quantification of the estimated prevalence and sensitivity. This situation is common in the risk analysis of potential forest pests where information is poor and in some cases even the native distribution of the organism is not clearly defined. To overcome this problem the use of mechanistic models, that link physiological characteristics with habitat occupation provide an alternative approach [67]–[68].

The incorporation of these approaches in plant health management will help planning and design of activities aimed at preventing establishment of pest species and improving phytosanitary status of forestry and agriculture in developing countries.

## Supporting Information

Figure S1 Projections of the fitted models into the 95% geographic kernel defined for *M. carolinensis*. Colors represent habitat suitability (0 = unsuitable, 1 = highly suitable). Red points correspond to the presence points used in the study.
(TIF)

Figure S2 Projections of the fitted models into the 95% geographic kernel defined for *M. marmorator*. Colors represent habitat suitability (0 = unsuitable, 1 = highly suitable). Red points correspond to the presence points used in the study.
(TIF)

Figure S3 Projections of the fitted models into the 95% geographic kernel defined for *M. notatus*. Colors represent habitat suitability (0 = unsuitable, 1 = highly suitable). Red points correspond to the presence points used in the study.
(TIF)

Figure S4 Projections of the fitted models into the 95% geographic kernel defined for *M. scutellatus*. Colors represent habitat suitability (0 = unsuitable, 1 = highly suitable). Red points correspond to the presence points used in the study.
(TIF)

Figure S5 Projections of the fitted models into the 95% geographic kernel defined for *M. titillator*. Colors represent habitat suitability (0 = unsuitable, 1 = highly suitable). Red points correspond to the presence points used in the study.
(TIF)

Figure S6 Projections of the fitted models into the 95% geographic kernel defined for *M. alternatus*. Colors represent habitat suitability (0 = unsuitable, 1 = highly suitable). Red points correspond to the presence points used in the study.
(TIF)

Figure S7 Projections of the fitted models into the 95% geographic kernel defined for *M. galloprovincialis*. Colors represent habitat suitability (0 = unsuitable, 1 = highly suitable). Red points correspond to the presence points used in the study.
(TIF)

Figure S8 Projections of the fitted models into the 95% geographic kernel defined for *M. saltuarius*. Colors represent habitat suitability (0 = unsuitable, 1 = highly suitable). Red points correspond to the presence points used in the study.
(TIF)

Figure S9 Projections of the fitted models into the 95% geographic kernel defined for *M. sutor*. Colors represent habitat suitability (0 = unsuitable, 1 = highly suitable). Red points correspond to the presence points used in the study.
(TIF)

Table S1 Geographic coordinates of the presence points used for each North American species.
(PDF)

Table S2 Geographic coordinates of the presence points used for each Eurasian species.
(PDF)
