# Supplementary figures and images for: Evaluating Habitat Suitability for the Establishment of Monochamus spp. through Climate-Based Niche Modeling
Source: PLoS One. 2014 Jul 14;9(7):e102592. doi: 10.1371/journal.pone.0102592 (PMC4097063; doi:10.1371/journal.pone.0102592)

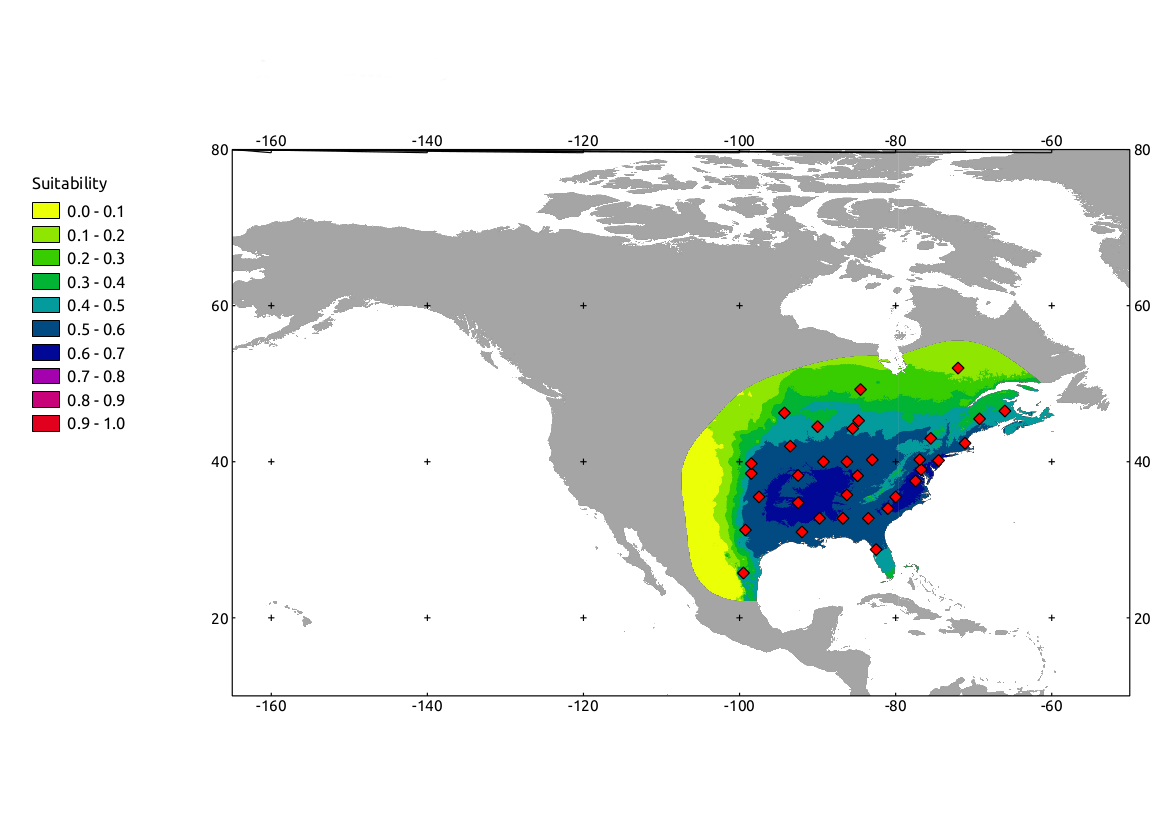

Supplement: Figure S1 — Projections of the fitted models into the 95% geographic kernel defined for M. carolinensis. Colors represent habitat suitability (0 = unsuitable, 1 = highly suitable). Red points correspond to the presence points used in the study. (TIF) [file pone.0102592.s001.tif]

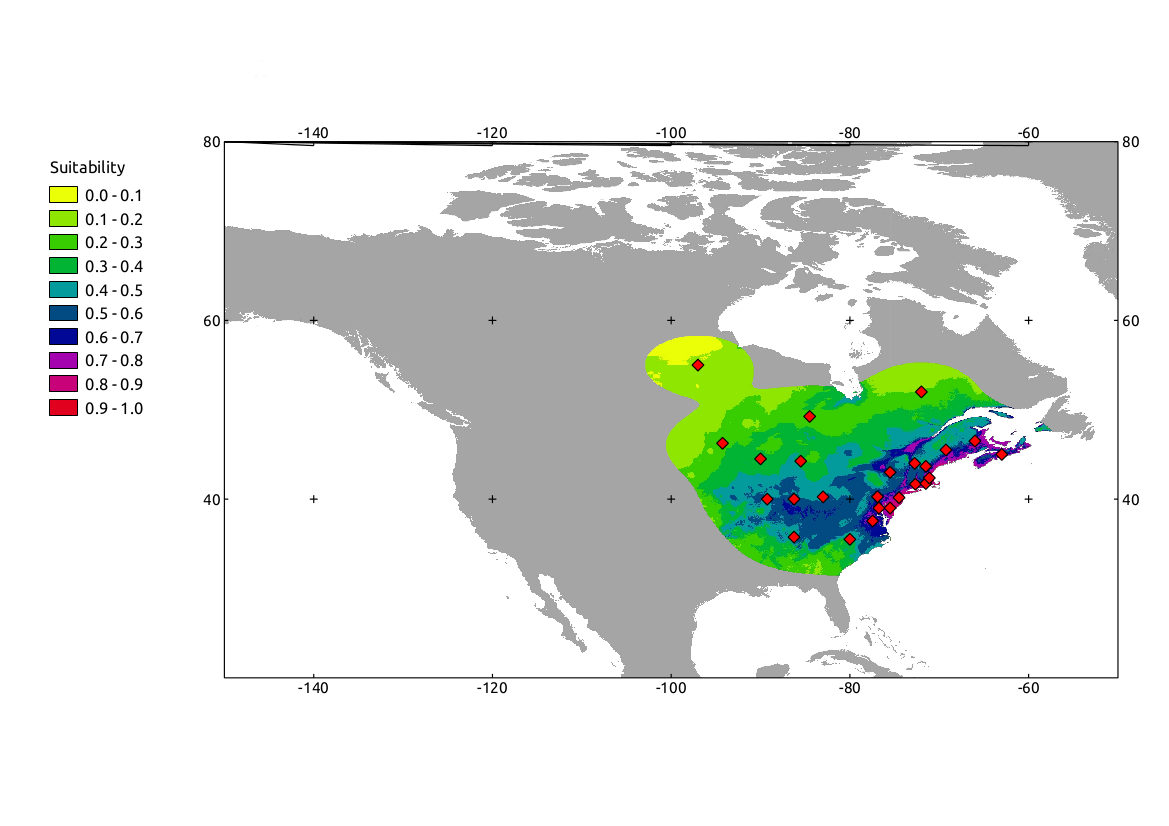

Supplement: Figure S2 — Projections of the fitted models into the 95% geographic kernel defined for M. marmorator. Colors represent habitat suitability (0 = unsuitable, 1 = highly suitable). Red points correspond to the presence points used in the study. (TIF) [file pone.0102592.s002.tif]

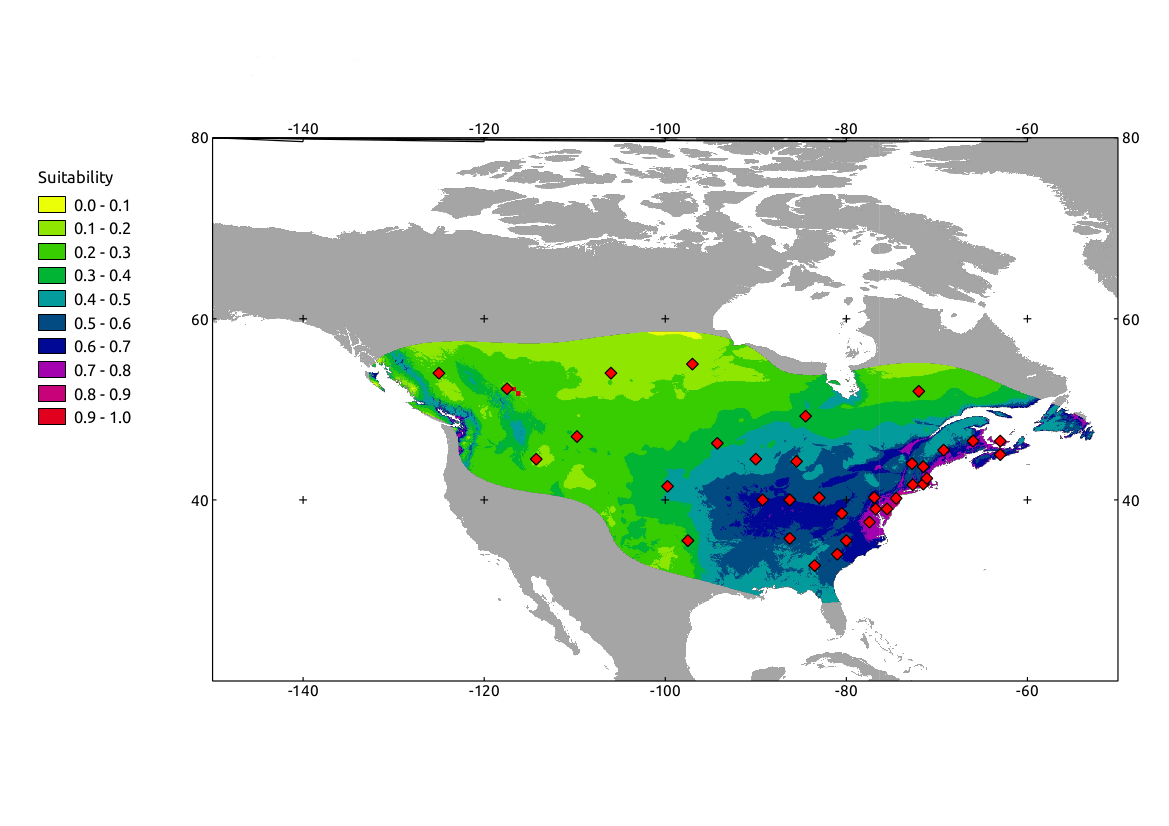

Supplement: Figure S3 — Projections of the fitted models into the 95% geographic kernel defined for M. notatus. Colors represent habitat suitability (0 = unsuitable, 1 = highly suitable). Red points correspond to the presence points used in the study. (TIF) [file pone.0102592.s003.tif]

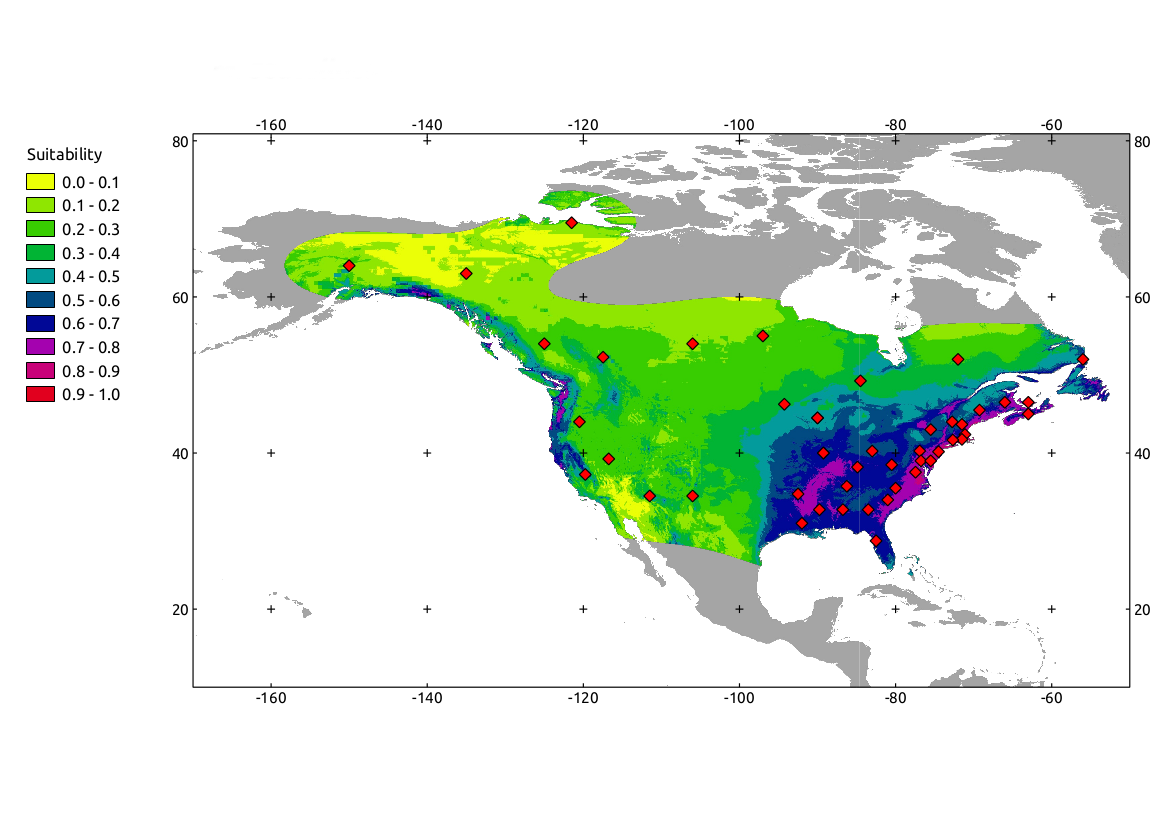

Supplement: Figure S4 — Projections of the fitted models into the 95% geographic kernel defined for M. scutellatus. Colors represent habitat suitability (0 = unsuitable, 1 = highly suitable). Red points correspond to the presence points used in the study. (TIF) [file pone.0102592.s004.tif]

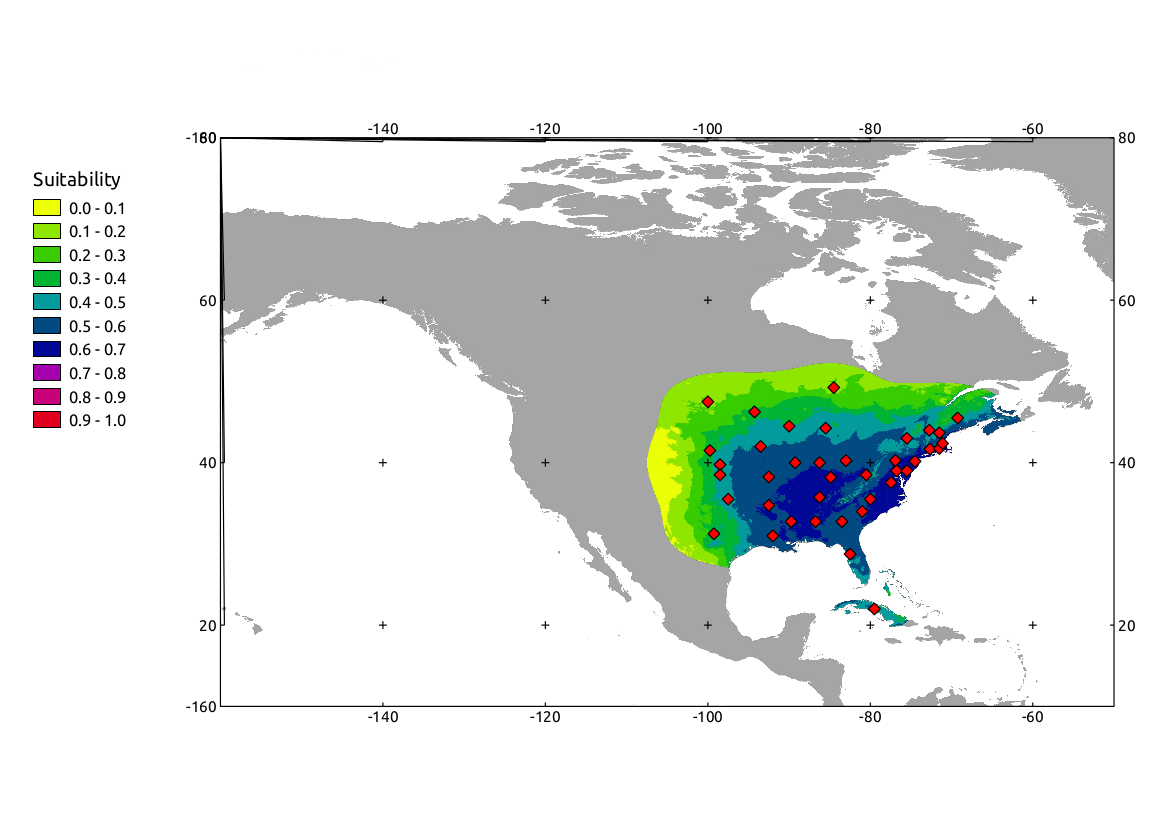

Supplement: Figure S5 — Projections of the fitted models into the 95% geographic kernel defined for M. titillator. Colors represent habitat suitability (0 = unsuitable, 1 = highly suitable). Red points correspond to the presence points used in the study. (TIF) [file pone.0102592.s005.tif]

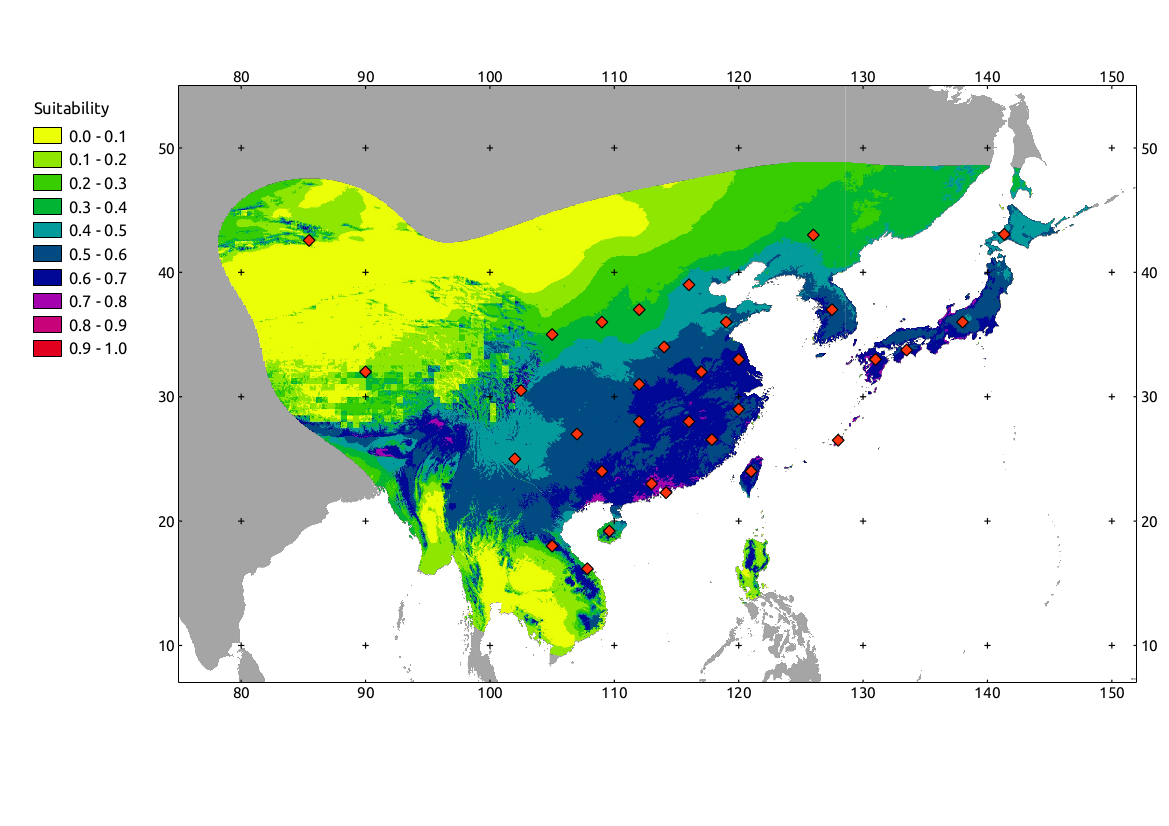

Supplement: Figure S6 — Projections of the fitted models into the 95% geographic kernel defined for M. alternatus. Colors represent habitat suitability (0 = unsuitable, 1 = highly suitable). Red points correspond to the presence points used in the study. (TIF) [file pone.0102592.s006.tif]

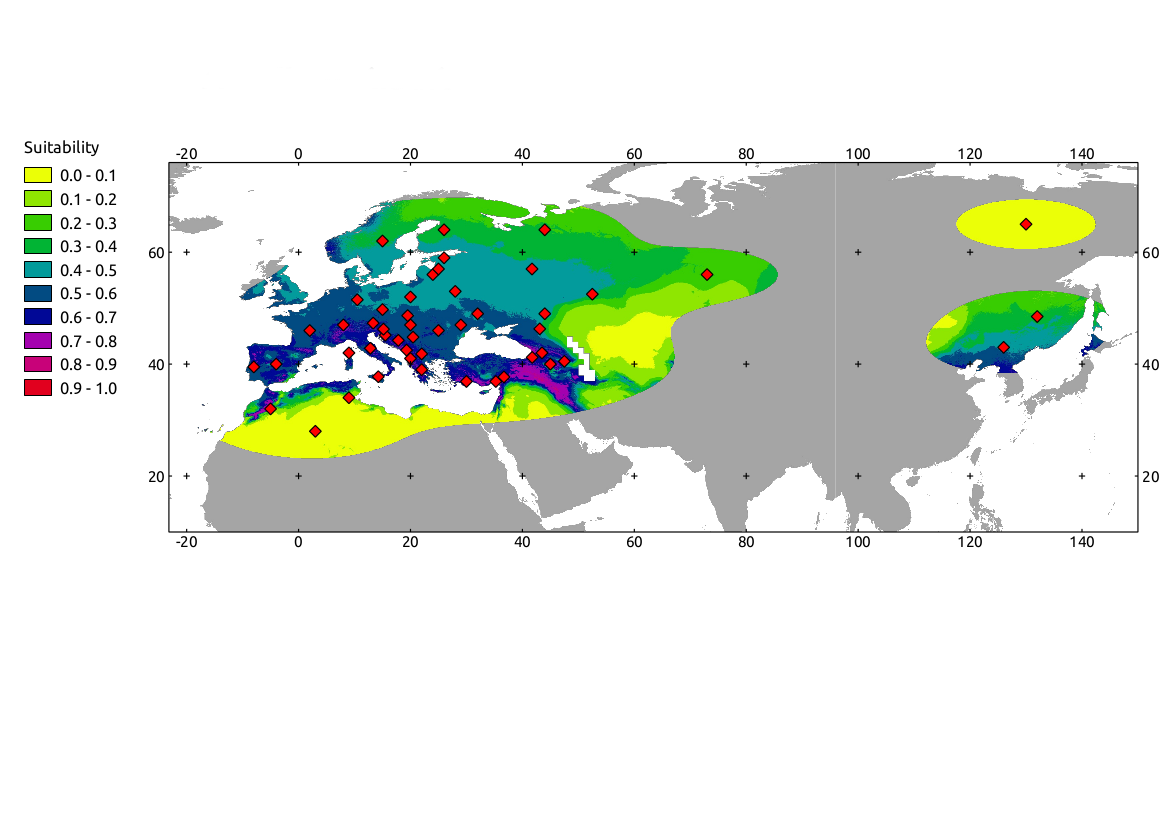

Supplement: Figure S7 — Projections of the fitted models into the 95% geographic kernel defined for M. galloprovincialis. Colors represent habitat suitability (0 = unsuitable, 1 = highly suitable). Red points correspond to the presence points used in the study. (TIF) [file pone.0102592.s007.tif]

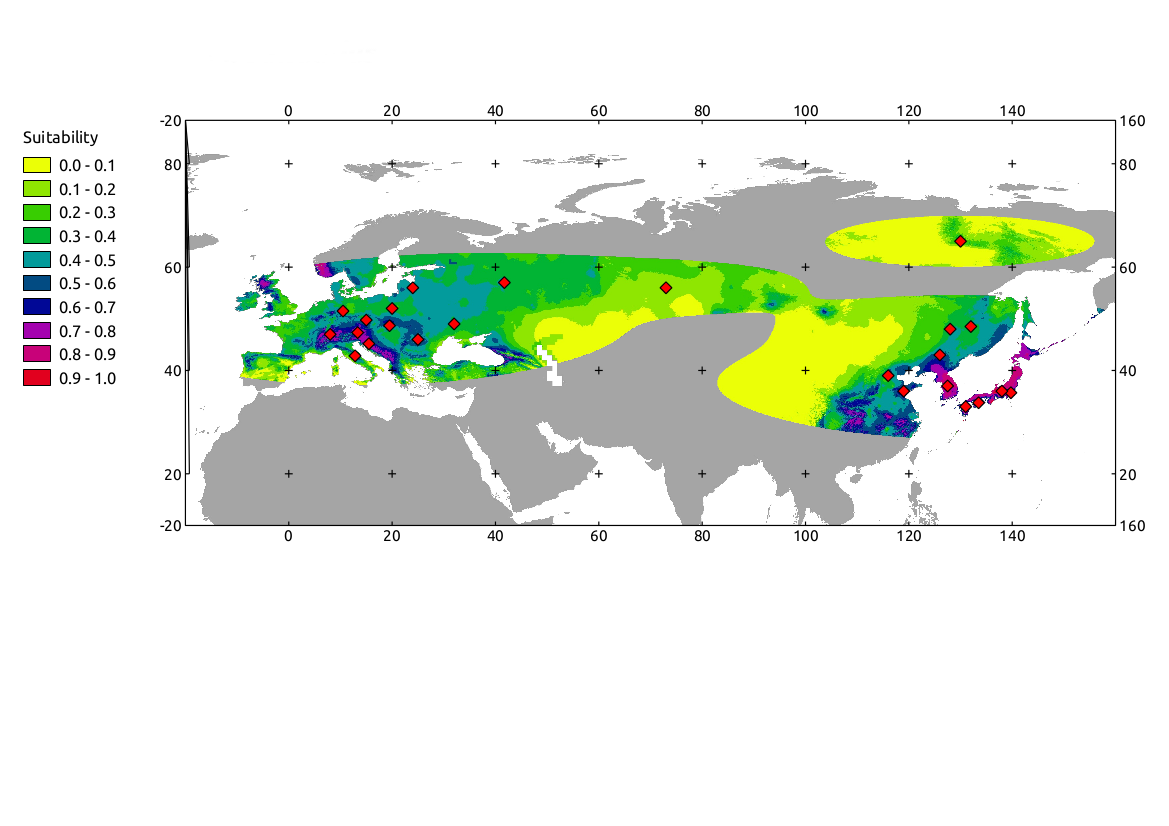

Supplement: Figure S8 — Projections of the fitted models into the 95% geographic kernel defined for M. saltuarius. Colors represent habitat suitability (0 = unsuitable, 1 = highly suitable). Red points correspond to the presence points used in the study. (TIF) [file pone.0102592.s008.tif]

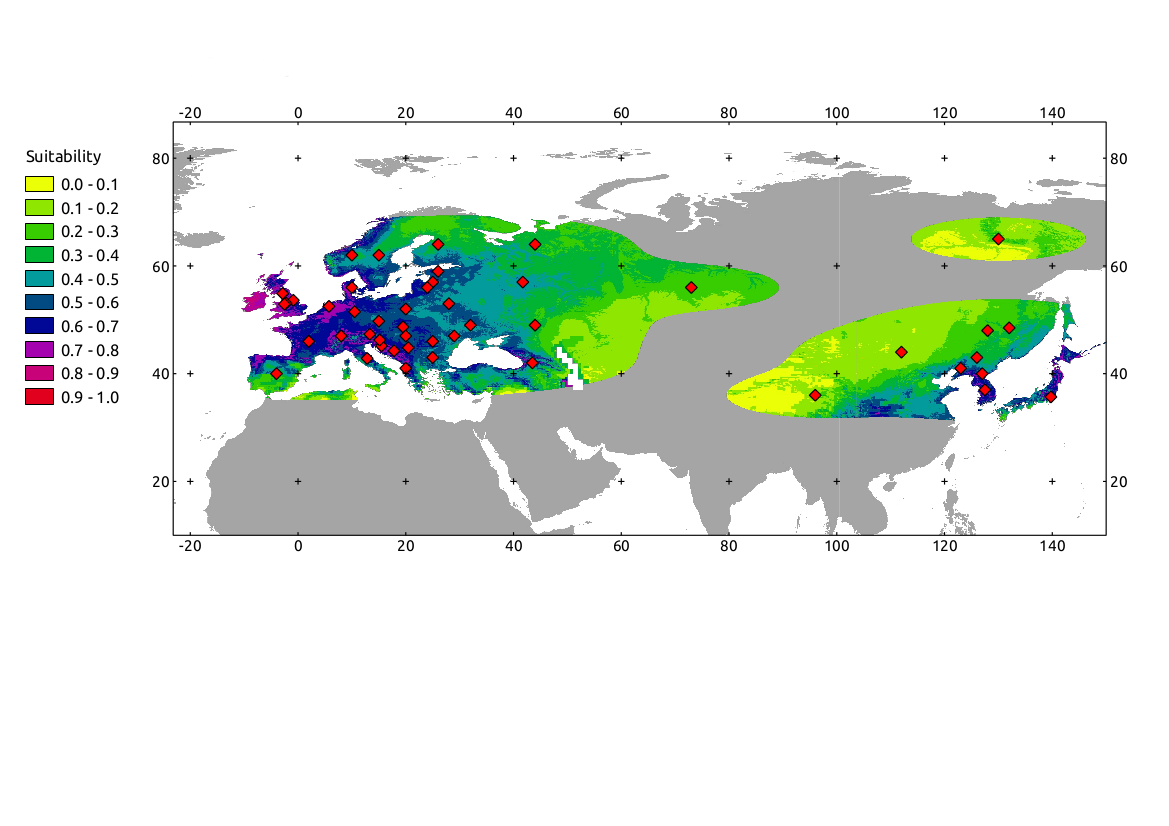

Supplement: Figure S9 — Projections of the fitted models into the 95% geographic kernel defined for M. sutor. Colors represent habitat suitability (0 = unsuitable, 1 = highly suitable). Red points correspond to the presence points used in the study. (TIF) [file pone.0102592.s009.tif]
