# Supplementary material for: Evaluating Habitat Suitability for the Establishment of Monochamus spp. through Climate-Based Niche Modeling
Source: PLoS One. 2014 Jul 14;9(7):e102592. doi: 10.1371/journal.pone.0102592 (PMC4097063; doi:10.1371/journal.pone.0102592)
Supplement: Table S1 — Geographic coordinates of the presence points used for each North American species. (PDF) [file pone.0102592.s010.pdf]

Table S1: Geographic coordinates of the presence points used for each North American species.

Species	Lat	Lon	Species	Lat	Lon	Species	Lat	Lon
M. carolinensis	25.75	-99.50	*M. notatus*	54.00	-106.00	*M. titillator*	37.55	-77.45
M. carolinensis	31.25	-99.25	*M. notatus*	55.00	-97.00	*M. titillator*	39.00	-75.50
M. carolinensis	35.50	-97.50	*M. notatus*	49.25	-84.50	*M. titillator*	40.17	-74.50
M. carolinensis	38.50	-98.50	*M. notatus*	52.00	-72.00	*M. titillator*	43.00	-75.50
M. carolinensis	39.76	-98.50	*M. notatus*	44.50	-114.25	*M. titillator*	41.67	-72.67
M. carolinensis	46.25	-94.25	*M. notatus*	47.00	-109.75	*M. titillator*	41.75	-71.50
M. carolinensis	42.00	-93.50	*M. notatus*	41.50	-99.75	*M. titillator*	42.37	-71.11
M. carolinensis	38.25	-92.50	*M. notatus*	35.50	-97.50	*M. titillator*	43.67	-71.50
M. carolinensis	34.75	-92.50	*M. notatus*	46.25	-94.25	*M. titillator*	44.00	-72.75
M. carolinensis	31.00	-92.00	*M. notatus*	44.50	-90.00	*M. titillator*	45.50	-69.25
M. carolinensis	44.50	-90.00	*M. notatus*	40.00	-89.25	*M. titillator*	49.25	-84.50
M. carolinensis	40.00	-89.25	*M. notatus*	44.25	-85.50	*M. titillator*	38.20	-84.88
M. carolinensis	32.75	-89.75	*M. notatus*	40.00	-86.25	*M. titillator*	39.00	-76.75
M. carolinensis	44.25	-85.50	*M. notatus*	35.75	-86.25	*M. scutellatus*	64.00	-150.00
M. carolinensis	40.00	-86.25	*M. notatus*	32.75	-83.50	*M. scutellatus*	63.00	-135.00
M. carolinensis	40.25	-83.00	*M. notatus*	34.00	-81.00	*M. scutellatus*	69.50	-121.50
M. carolinensis	38.20	-84.88	*M. notatus*	35.50	-80.00	*M. scutellatus*	54.00	-125.00
M. carolinensis	35.75	-86.25	*M. notatus*	38.50	-80.50	*M. scutellatus*	52.28	-117.47
M. carolinensis	32.75	-86.75	*M. notatus*	40.25	-83.00	*M. scutellatus*	54.00	-106.00
M. carolinensis	28.75	-82.50	*M. notatus*	40.27	-76.91	*M. scutellatus*	55.00	-97.00
M. carolinensis	32.75	-83.50	*M. notatus*	39.00	-76.75	*M. scutellatus*	49.25	-84.50
M. carolinensis	34.00	-81.00	*M. notatus*	37.55	-77.45	*M. scutellatus*	52.00	-72.00
M. carolinensis	35.50	-80.00	*M. notatus*	39.00	-75.50	*M. scutellatus*	52.00	-56.00
M. carolinensis	37.55	-77.45	*M. notatus*	40.17	-74.50	*M. scutellatus*	44.00	-120.50
M. carolinensis	39.00	-76.75	*M. notatus*	41.67	-72.67	*M. scutellatus*	39.25	-116.75
M. carolinensis	40.27	-76.91	*M. notatus*	41.75	-71.50	*M. scutellatus*	37.25	-119.75
M. carolinensis	43.00	-75.50	*M. notatus*	42.37	-71.11	*M. scutellatus*	34.50	-111.50
M. carolinensis	40.17	-74.50	*M. notatus*	43.67	-71.50	*M. scutellatus*	34.50	-106.00
M. carolinensis	42.37	-71.11	*M. notatus*	44.00	-72.75	*M. scutellatus*	46.25	-94.25
M. carolinensis	45.50	-69.25	*M. notatus*	45.50	-69.25	*M. scutellatus*	44.50	-90.00
M. carolinensis	46.50	-66.00	*M. notatus*	46.50	-66.00	*M. scutellatus*	40.00	-89.25
M. carolinensis	52.00	-72.00	*M. notatus*	46.50	-63.00	*M. scutellatus*	34.75	-92.50
M. carolinensis	49.25	-84.50	*M. notatus*	45.00	-63.00	*M. scutellatus*	31.00	-92.00
M. carolinensis	45.26	-84.75	*M. notatus*	40.00	-86.25	*M. scutellatus*	32.75	-89.75
M. marmorator	55.00	-97.00	*M. titillator*	22.00	-79.50	*M. scutellatus*	44.50	-90.00
M. marmorator	46.25	-94.25	*M. titillator*	28.75	-82.50	*M. scutellatus*	40.00	-89.25
M. marmorator	44.50	-90.00	*M. titillator*	47.50	-100.00	*M. scutellatus*	40.25	-83.00
M. marmorator	40.00	-89.25	*M. titillator*	41.50	-99.75	*M. scutellatus*	38.20	-84.88
M. marmorator	35.75	-86.25	*M. titillator*	39.76	-98.50	*M. scutellatus*	35.75	-86.25
M. marmorator	49.25	-84.50	*M. titillator*	38.50	-98.50	*M. scutellatus*	32.75	-86.75
M. marmorator	44.25	-85.50	*M. titillator*	35.50	-97.50	*M. scutellatus*	38.50	-80.50
M. marmorator	40.00	-86.25	*M. titillator*	31.25	-99.25	*M. scutellatus*	35.50	-80.00
M. marmorator	40.25	-83.00	*M. titillator*	46.25	-94.25	*M. scutellatus*	34.00	-81.00
M. marmorator	35.50	-80.00	*M. titillator*	42.00	-93.50	*M. scutellatus*	32.75	-83.50
M. marmorator	37.55	-77.45	*M. titillator*	38.25	-92.50	*M. scutellatus*	28.75	-82.50
M. marmorator	39.00	-76.75	*M. titillator*	34.75	-92.50	*M. scutellatus*	40.27	-76.91
M. marmorator	40.27	-76.91	*M. titillator*	31.00	-92.00	*M. scutellatus*	39.00	-76.75
M. marmorator	39.00	-75.50	*M. titillator*	44.50	-90.00	*M. scutellatus*	37.55	-77.45
M. marmorator	40.17	-74.50	*M. titillator*	40.00	-89.25	*M. scutellatus*	39.00	-75.50
M. marmorator	43.00	-75.50	*M. titillator*	32.75	-89.75	*M. scutellatus*	40.17	-74.50
M. marmorator	41.67	-72.67	*M. titillator*	44.25	-85.50	*M. scutellatus*	43.00	-75.50
M. marmorator	41.75	-71.50	*M. titillator*	40.00	-86.25	*M. scutellatus*	44.00	-72.75

M. marmorator	42.37	-71.11	*M. titillator*	35.75	-86.25	*M. scutellatus*	43.67	-71.50
M. marmorator	43.67	-71.50	*M. titillator*	32.75	-86.75	*M. scutellatus*	42.37	-71.11
M. marmorator	44.00	-72.75	*M. titillator*	40.25	-83.00	*M. scutellatus*	41.75	-71.50
M. marmorator	45.50	-69.25	*M. titillator*	32.75	-83.50	*M. scutellatus*	41.67	-72.67
M. marmorator	46.50	-66.00	*M. titillator*	34.00	-81.00	*M. scutellatus*	45.50	-69.25
M. marmorator	45.00	-63.00	*M. titillator*	35.50	-80.00	*M. scutellatus*	46.50	-66.00
M. marmorator	52.00	-72.00	*M. titillator*	38.50	-80.50	*M. scutellatus*	46.50	-63.00
M. notatus	54.00	-125.00	*M. titillator*	40.27	-76.91	*M. scutellatus*	45.00	-63.00
M. notatus	52.28	-117.47						
