# Supplementary material for: Evaluating Habitat Suitability for the Establishment of Monochamus spp. through Climate-Based Niche Modeling
Source: PLoS One. 2014 Jul 14;9(7):e102592. doi: 10.1371/journal.pone.0102592 (PMC4097063; doi:10.1371/journal.pone.0102592)
Supplement: Table S2 — Geographic coordinates of the presence points used for each Eurasian species. (PDF) [file pone.0102592.s011.pdf]

Table S2: Geographic coordinates of the presence points used for each Eurasian species.

Species	*Lat*	*Lon*
M. alternatus	42.57	85.46
M. alternatus	32.00	90.00
M. alternatus	16.17	107.83
M. alternatus	18.00	105.00
M. alternatus	19.20	109.60
M. alternatus	35.00	105.00
M. alternatus	30.50	102.50
M. alternatus	25.00	102.00
M. alternatus	27.00	107.00
M. alternatus	24.00	109.00
M. alternatus	36.00	109.00
M. alternatus	37.00	112.00
M. alternatus	34.00	114.00
M. alternatus	31.00	112.00
M. alternatus	28.00	112.00
M. alternatus	23.00	113.00
M. alternatus	22.29	114.16
M. alternatus	39.00	116.00
M. alternatus	36.00	119.00
M. alternatus	32.00	117.00
M. alternatus	28.00	116.00
M. alternatus	26.55	117.84
M. alternatus	29.00	120.00
M. alternatus	33.00	120.00
M. alternatus	24.00	121.00
M. alternatus	43.00	126.00
M. alternatus	37.00	127.50
M. alternatus	26.50	128.00
M. alternatus	33.00	131.00
M. alternatus	33.75	133.50
M. alternatus	36.00	138.00
M. alternatus	43.06	141.35
M. sutor	35.69	139.75
M. sutor	37.00	127.50
M. sutor	40.00	127.00
M. sutor	41.00	123.00
M. sutor	43.00	126.00
M. sutor	48.00	128.00
M. sutor	48.50	132.00
M. sutor	65.00	130.00
M. sutor	44.00	112.00
M. sutor	36.00	96.00
M. sutor	56.00	73.00
M. sutor	64.00	44.00
M. sutor	57.00	41.72
M. sutor	49.00	44.00
M. sutor	42.00	43.50
M. sutor	64.00	26.00
M. sutor	59.00	26.00
M. sutor	57.00	25.00
M. sutor	56.00	24.00
M. sutor	53.00	28.00
M. sutor	49.00	32.00
M. sutor	47.00	29.00
M. sutor	46.00	25.00
M. sutor	43.00	25.00
M. sutor	52.00	20.00
M. sutor	48.67	19.50
M. sutor	47.00	20.00
M. sutor	44.82	20.46
M. sutor	41.00	20.00
M. sutor	44.25	17.83
M. sutor	45.17	15.50
M. sutor	46.25	15.17
M. sutor	47.33	13.33
M. sutor	49.75	15.00
M. sutor	42.83	12.83
M. sutor	40.00	-4.00
M. sutor	46.00	2.00
M. sutor	47.00	8.01
M. sutor	52.50	5.75
M. sutor	51.50	10.50
M. sutor	56.00	10.00
M. sutor	62.00	10.00
M. sutor	62.00	15.00
M. sutor	53.63	-0.87
M. sutor	53.09	-2.22
M. sutor	52.91	-2.52
M. sutor	54.89	-2.86
M. saltuarius	47.33	13.33
M. saltuarius	49.75	15.00
M. saltuarius	45.17	15.50
M. saltuarius	48.67	19.50
M. saltuarius	52.00	20.00
M. saltuarius	56.00	24.00
M. saltuarius	46.00	25.00
M. saltuarius	49.00	32.00
M. saltuarius	57.00	41.72
M. saltuarius	56.00	73.00
M. saltuarius	39.00	116.00
M. saltuarius	36.00	119.00
M. saltuarius	65.00	130.00
M. saltuarius	48.50	132.00
M. saltuarius	48.00	128.00
M. saltuarius	43.00	126.00
M. saltuarius	37.00	127.50
M. saltuarius	33.00	131.00
M. saltuarius	33.75	133.50
M. saltuarius	35.69	139.75
M. saltuarius	36.00	138.00
M. saltuarius	51.50	10.50
M. saltuarius	47.00	8.01
M. saltuarius	42.83	12.83
M. galloprovincialis	36.90	30.00
M. galloprovincialis	36.90	35.25
M. galloprovincialis	37.70	36.70
M. galloprovincialis	41.20	41.75
M. galloprovincialis	46.30	43.10
M. galloprovincialis	28.00	3.00
M. galloprovincialis	34.00	9.00
M. galloprovincialis	40.00	-4.00
M. galloprovincialis	40.50	47.50
M. galloprovincialis	42.00	9.00
M. galloprovincialis	42.83	12.83
M. galloprovincialis	43.00	126.00
M. galloprovincialis	44.25	17.83
M. galloprovincialis	44.82	20.46
M. galloprovincialis	45.17	15.50
M. galloprovincialis	46.25	15.17
M. galloprovincialis	47.00	20.00
M. galloprovincialis	49.00	44.00
M. galloprovincialis	49.75	15.00
M. galloprovincialis	51.50	10.50
M. galloprovincialis	52.50	52.50
M. galloprovincialis	57.00	41.72
M. galloprovincialis	59.00	26.00
M. galloprovincialis	65.00	130.00
M. galloprovincialis	32.00	-5.00
M. galloprovincialis	37.75	14.25
M. galloprovincialis	39.00	22.00
M. galloprovincialis	39.50	-8.00
M. galloprovincialis	40.00	45.00
M. galloprovincialis	41.00	20.00
M. galloprovincialis	41.83	22.00
M. galloprovincialis	42.00	43.50
M. galloprovincialis	42.50	19.30
M. galloprovincialis	46.00	2.00
M. galloprovincialis	46.00	25.00
M. galloprovincialis	47.00	29.00
M. galloprovincialis	47.00	8.01
M. galloprovincialis	47.33	13.33
M. galloprovincialis	48.50	132.00
M. galloprovincialis	48.67	19.50
M. galloprovincialis	49.00	32.00
M. galloprovincialis	52.00	20.00
M. galloprovincialis	53.00	28.00
M. galloprovincialis	56.00	24.00
M. galloprovincialis	56.00	73.00
M. galloprovincialis	57.00	25.00
M. galloprovincialis	62.00	15.00
M. galloprovincialis	64.00	44.00
M. galloprovincialis	64.00	26.00
